# Automated Battery Making Fault Classification Using Over-Sampled Image Data CNN Features

**DOI:** 10.3390/s23041927

**Published:** 2023-02-08

**Authors:** Nasir Ud Din, Li Zhang, Yatao Yang

**Affiliations:** College of Electronics and Information Engineering, Shenzhen University, Shenzhen 518060, China

**Keywords:** fault detection, deep learning, machine learning, SMOTE, image classification

## Abstract

Due to the tremendous expectations placed on batteries to produce a reliable and secure product, fault detection has become a critical part of the manufacturing process. Manually, it takes much labor and effort to test each battery individually for manufacturing faults including burning, welding that is too high, missing welds, shifting, welding holes, and so forth. Additionally, manual battery fault detection takes too much time and is extremely expensive. We solved this issue by using image processing and machine learning techniques to automatically detect faults in the battery manufacturing process. Our approach will reduce the need for human intervention, save time, and be easy to implement. A CMOS camera was used to collect a large number of images belonging to eight common battery manufacturing faults. The welding area of the batteries’ positive and negative terminals was captured from different distances, between 40 and 50 cm. Before deploying the learning models, first, we used the CNN for feature extraction from the image data. To over-sample the dataset, we used the Synthetic Minority Over-sampling Technique (SMOTE) since the dataset was highly imbalanced, resulting in over-fitting of the learning model. Several machine learning and deep learning models were deployed on the CNN-extracted features and over-sampled data. Random forest achieved a significant 84% accuracy with our proposed approach. Additionally, we applied K-fold cross-validation with the proposed approach to validate the significance of the approach, and the logistic regression achieved an 81.897% mean accuracy score and a +/− 0.0255 standard deviation.

## 1. Introduction

Battery technology converts chemical energy into an electrical current so that energy can be stored for later use. In a battery, the cathode and anode are submerged in an electrolyte, which is a chemical solution. Positive and negative terminals connect the anode and cathode of a battery. When the positive and negative terminals of a battery are linked, an electric current is generated and flows throughout the battery [1]. Positive terminals are characterized by their ability to accept electrons from another electrode [2]. Battery terminals made of good-quality materials and manufactured with good skill can increase battery life. The important faults in the battery making process are burning the positive or negative terminals, welding too high, the wrong welding, welding holes, a lack of welding, the wrong cover, continuous holes, and shifting the terminals. These faults will lead to huge losses for companies if they are not tested accurately and on time. To provide a high-quality product to the customers, it is essential to detect any faults in the battery manufacturing phase [3]. Welding technology has become more commonplace in the production of batteries [4]. Automated systems are performing welding at big plants, but the quality of the products is not being ensured. To ensure the quality of the batteries, companies perform manual inspections of terminals so they can track the faulty batteries. This manual inspection is too expensive and time consuming. In response to the problems associated with the manual inspection of defects [5], automatic detection techniques such as pattern recognition and machine learning are mainly used [6,7,8]. Machine-vision-based defect detection systems can also be deployed to detect faults in batteries.

The detection of manufacturing faults in batteries is crucial to enhance safety precautions. Batteries are overtaking other energy storage systems in many contexts, including electric vehicles, grid electricity storage, and a variety of electronic devices, due to their superior compactness, power density, long life, low toxicity, and low self-discharge rate [9]. As with other high-energy storage devices, batteries provide some danger, and there is always the possibility of faults in manufacturing design or material distribution. The working properties of the battery system are extremely obscure. Single cells in battery systems can be connected in series, parallel, or series–parallel arrangements to fulfill the vehicle’s mileage and traction power requirements [10]. During the manufacturing process, however, the battery cell itself is initially inconsistent. A different location for the battery cell in the system will result in a different working environment, which will exacerbate the initial inconsistency and eventually lead to overcharging, overdischarging, or internal short-circuit (ISC) faults of individual cells in the system, which will hinder the battery system’s normal operation [11].

The battery faults and warning signs were detected by Xiong et al. [12] using a rule-based and probabilistic-based method. Errors in the real-time monitoring system, both at room and high temperatures, were predicted by their analysis. Muddappa and Anwar [13] utilized a method based on fuzzy logic to detect a variety of battery issues. The results indicated that the proposed method can detect several fault classes, including overcharge, overdischarge, and aging of the battery quickly and reliably. As a result, it provides an effective and precise way of detecting faults in Li-ion batteries. Yao et al. [14] utilized an intelligent-based method for lithium battery fault diagnosis. First, they employed the discrete cosine filter technique to acquire sufficient de-noising, and then, they applied the covariance matrix of filtered data for current fluctuations. Thirdly, a Support Vector Machine (SVM) with grid search was utilized to detect faults. Yang et al. [15] used a Visual-Geometry-Group (VGG)-based deep learning model for defect classification and inspection of the welding quality of lasers. The model was trained with over 8000 images. Their results showed that the VGG model accurately classified the defects. The welding defects of battery safety vents were proposed to be detected using a lightweight and effective deep learning algorithm. A huge number of images were collected for the binary and seven-class classification tasks. The proposed model was compared to six other CNN models [16].

Following the important research domain, this study also worked on battery making faults. The first step in our approach was to capture images of different battery faults in order to build a dataset. We used a deep learning model to extract the features and then trained several models for the prediction of faults. The key points of this study are as follows:

- This study collected a dataset from the battery manufacturing plant using a high-megapixel CMOS digital camera with a white LED annular source.
- The collected dataset was imbalanced, and to overcome this problem, this study used the Synthetic Minority Over-sampling Technique (SMOTE) on the image features to generate a sample for the minority class.
- A deep learning model Convolutional Neural Networks (CNNs) was deployed with fine-tuned architecture to extract the features.
- Several machine learning and deep learning models were evaluated using the CNN features and SMOTE over-sampled dataset.

The remainder of the paper is structured as follows: Section 2 discusses various relevant research studies. Section 3 describes the data collection, preprocessing, dataset balancing, a methodological approach, machine learning, deep learning models, and the evaluation metrics. Section 4 includes the results and discussions. Section 5 is the conclusions.

## 2. Literature Review

Currently, researchers all over the world are performing research on battery faults to improve safety measures and the life of products by detecting the various faults in battery systems. For example, Chen et al. [17] used a two-layer-based model for battery fault diagnosis. A fault diagnosis approach was presented in a paper [18] to detect thermal faults in the battery cell. The two-state thermal model was used to capture the core and surface. Liu et al. [19] utilized a sensor-based defect detection technique for batteries, and an adaptive extended Kalman filter was applied to assist in the generation of the residual. Then, a statistical inference method was used to figure out if the fault existed or not based on the residuals.

The researchers Adnan et al. [20] proposed a new data-driven method for embedded diagnostics and predictions of battery health using the machine learning classifier SVM for training and testing data that were preprocessed based on the load. Ardeshiri et al. [21] first analyzed the battery management system limitations and problems, and then, they analyzed the components that could affect the performance. Second, they created a model for the battery faults; it is very difficult to determine the internal features, health, and charging capacity with machine learning models. Logistic Regression (LR) was the simplest and most-effective model. It was determined that Kernel Support Vector Machine (KSVM) performed better than KNN in terms of accuracy while classifying the data.

The study also compared the different models to evaluate the pros and cons of each approach. The wavelet features of voltage correlations were used by the research in [22] to automatically evaluate battery packs connected in series for defects. In order to locate the abnormal electrical signals, they first analyzed the cross-cell voltages of several cells using a refined recursive Pearson correlation coefficient and reconstructed fault-related features from wavelet sub-bands; the coefficient series was next deconstructed from a wavelet packet. A multi-classification Relevance Vector Machine (mRVM) and an Artificial Neural Network (ANN) were used to analyze the fault mechanisms and their effects. A multi-classification relevance vector machine was then used to examine the ANN’s failure mode and fault degree classifications (mRVM). The proposed method identified and located various faults in an experimental setting. mRVM performed better at finding thermal faults than the ANN; although mRVM was more accurate in some conditions, the ANN had a higher overall diagnostic performance. The internal short-circuit of the battery was detected and identified using the SVM approach by Sabri et al. [23]. When it was difficult to obtain the faulty battery data, SVM and anomaly detection offered a good alternative for fault detection. The battery current and voltage were employed as features to detect the short-circuit. The proposed method offers excellent fault detection accuracy in both training and testing. Hariharan et al. [24] used a Random Forest (RF) model to detect internal battery faults. The features for the training set were prepared with and without external short-circuits between the battery terminals. The RF model was tested with high accuracy after being trained on a training set.

Currently, some voltage-based battery fault detection methods are being investigated in order to reflect the faults at all battery terminals. Consequently, entropy has been used to assess and evaluate uncertainty. The Shannon entropy was used to design a voltage fault detection method for detecting battery faults [25]. These approaches were only utilized to identify battery faults and their levels; they were unable to detect faults such as abnormal battery current and temperature in a timely manner and may have missed these certain faults [17]. In electric vehicles, fault detection is crucial to the effective running of the batteries. The Empirical Mode Decomposition (EMD) approach was initially used to extract features from batteries by decomposing the battery voltage signals and reducing noise during this process. The collected features were then utilized to calculate the sample entropy values for fault detection [26]. To overcome this problem, a battery terminal fault detection approach based on the correlation coefficient was proposed.

In the literature review, most researchers have focused on the fault detection of battery systems that contain internal and external faults such as overcharging, discharging, internal short-circuits, battery health faults, charging capacity, voltage, and thermal faults. These faults were primarily detected using rule-based, signal processing, and entropy-based techniques; being less accurate and time-consuming. Machine learning was used in some studies to detect battery faults. To the best of our knowledge, the fault dataset we used to detect faults has not been used by any other study with the machine and deep learning models. The summary of the literature review is given in Table 1.

## 3. Materials and Methods

**Figure 1:**
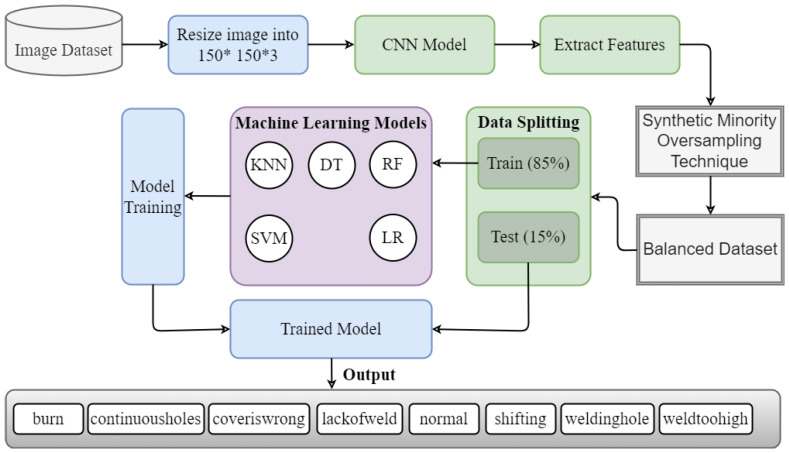
Proposed workflow diagram for battery fault detection.

This study performed experiments for battery fault detection using supervised machine learning. First, we collected the image dataset using a CMOS camera and then extracted the features using the CNN model. After feature extraction, the SMOTE was used to resolve the data imbalance problem. Data splitting was performed after data balancing with an 85:15 ratio, where 85% of the data were used for the training of the models and 15% for the testing of the models. We evaluated all models in terms of accuracy, precision, recall, and F1 score. The proposed workflow for battery fault detection is shown in Figure 1.

### 3.1. Dataset Description and Preprocessing

**Figure 2:**
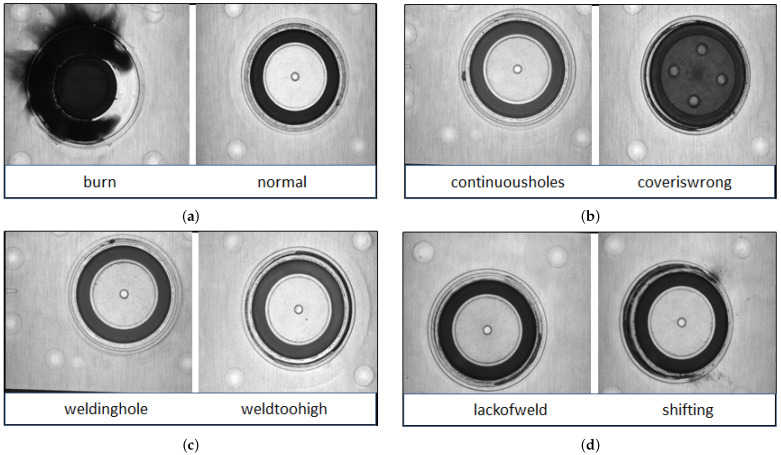
Sample of battery fault images: (**a**) the right side shows the normal image and the left side shows the burn image; (**b**) the right side shows the cover is the wrong image, and the left side shows the image of the continuous hole; (**c**) the right side shows the weld too high image, and the left side shows the welding hole image; (**d**) right side shows the shifting image, and the left side shows the lack of weld image.

A complementary metal–oxide semiconductor (CMOS) camera was used to capture an image of the welding area, which was illuminated by an annular white LED with a brightness range of 0–255. Compared to CMOS cameras, the cost of the CCD camera used in an industrial system is higher. In manufacturing environments, the CMOS camera is widely used due to its high image quality. As part of this study, we used a 5-megapixel industrial camera to obtain extraordinarily detailed images of the welding areas. As a result, a 2D grey image may accurately depict the 3D geometric information of the welding area. The welded area was more easily visible owing to a white annular light source, which was focused on the object at an angle of about 90 degrees. When the camera was active, white LED light beams were applied to the surface of the battery and reflected back into the device. We shot from a variety of distances, averaging between 40 and 50 cm. The intensity of the light was also changed at random, with levels ranging from about 50 to 150. A total of 3736 images were collected, which belonged to 8 classes. There were 879 images for continuous holes, 840 images for welding holes, 466 images for normal, 711 images for weld too high, 290 images for lack of welding, 232 images for shifting, 126 images for burn, and 192 images for cover is wrong. The images had different heights and widths. The deep learning models took only fixed-size images for training and testing. We resized the image to 150 × 150 × 3. The samples were taken from the battery-making faults dataset and are shown in Figure 2.

### 3.2. SMOTE and Splitting

The collected dataset was imbalanced, containing 232 shifting images, 192 cover is wrong images, and 126 burn images, as compared to 879 continuous hole images. The burn class was highly imbalanced, with only 3% of the whole dataset. The imbalanced dataset problem can cause machine learning models over-fitting toward the majority class data. To resolve this issue, we applied the SMOTE, which automatically generates new artificial samples from the existing images by selecting those that are close to the feature space [28,29]. The SMOTE over-sampled the minority classes with new samples. After deploying the SMOTE, we split the battery making faults dataset into 85% training and 15% testing to detect the faults and evaluate the model’s performance. Table 2 represents the training and testing images.

### 3.3. Convolutional Neural Network Architecture for Feature Extraction

**Figure 3:**
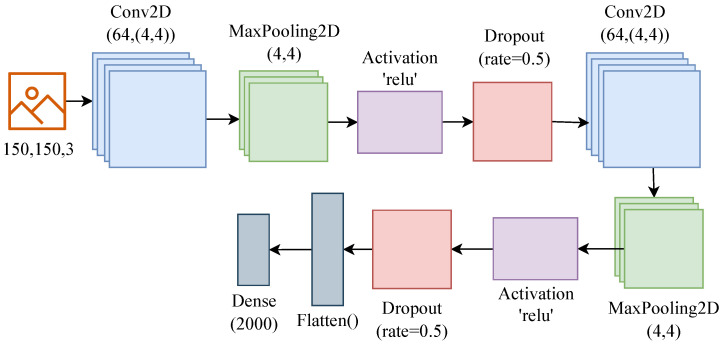
CNN architecture for feature extraction.

In deep learning algorithms, a CNN is a kind of network architecture that is used specifically for tasks such as image recognition [30]. The CNN is extensively used in disease diagnosis [31], speech recognition [32], object classification [33], and fault detection [34]. A CNN needs to simplify the images without removing important details in order to provide reliable results [35]. CNNs classify images using specialized convolution and pooling operations while being computationally efficient. In a conventional CNN, there are four layers: (1) a convolution layer; (2) a pooling layer; (3) an activation layer; (4) a fully connected layer with a linearly corrected activation [36]. We used the CNN for feature extraction from the images [37], and the architecture of the used CNN model is represented in Figure 3. The input image size for the CNN was 150 × 150 × 3, followed by a 4 × 4 pooling size [38], Rectified Linear Activation Function (ReLU), and a 0.5 dropout rate [38]. These same layers’ patterns repeated two times made the CNN architecture deeper to extract the significant features. Following the CNN layers, we used the flatten layer to convert the 2D data into 1D [36], and in the end, we used a dense layer with 200 neurons, which will help to extract 2000 features from each image.

### 3.4. Machine Learning Models

Artificial Intelligence (AI) entails machine learning, the ability of machines to identify patterns from the input data and make predictions from unseen data with little human involvement. The machine learns to maximize its performance to produce better outcomes from large datasets. By utilizing machines’ decision-making abilities, it is possible to abstract the results from a large dataset with little effort. A wide variety of machine learning applications can be found in different domains, such as the Internet of Things (IoT) [39], health care [40], machine vision [41], edge computing [42], security [43,44], and many others.

In this study, we used several machine learning models to predict battery faults. We took the last layer of the CNN as the input for the machine learning models. We used a dense layer at the end of the CNN with 2048 neurons. Therefore, the input for the machine learning models will be 2048 features when we use the CNN for the feature extraction. The hyper-parameters for machine learning are presented in Table 3.

#### 3.4.1. Support Vector Machine

SVM is an excellent supervised model for image classification because it can separate data into distinct classes. When compared to CNN’s model, SVM’s classification capability is superior. The best results for image classification using SVM emerged from using a “poly” kernel. In the case of large datasets, the training time of SVM is long and complex. In order to classify the data, a hyperplane is identified that acts as a sharp dividing line between two classes. One way to find the optimal hyperplane is to find the one with the largest deviation between data points. This is in addition to choosing a plane that fairly divides the data into predetermined classes. Accordingly, the gap is the separation between two points. When comparing two hyperplanes, sometimes, the one with the largest margin is less important than the one with the best data separation. The SVM model is trained on the training set of data and then applied to the classification of the test data once a hyperplane is found that satisfies the data requirements [45].

#### 3.4.2. Logistic Regression

Logistic regression is a supervised ML model that can be constructed with core statistics to predict the probability of the target variables. Utilizing sigmoid functions, the corresponding probability is derived. Due to its efficacy in identifying defaulters, this ML technique has received significant recognition; in addition, it is one of the simplest to apply across a broad range of classification tasks [46,47]. Because of its improved flexibility and reduced amount of parameters, logistic regression succeeds at handling binary classification problems.

#### 3.4.3. Random Forest

A random forest is a method for classification that uses multiple decision trees to interpret the data. One of the most-popular machine learning algorithms, a random forest classifier does not require hyper-parameter tuning to produce accurate results. Since it is both easy to implement and flexible, random forest has become a popular machine learning preference. Known also as bagged decision trees, these trees use the bootstrap aggregation technique to build robust learners from a pool of relatively ineffective ones. The random forest classifier builds a new decision tree from scratch based on a sample of data randomly selected [48].

#### 3.4.4. Decision Tree

Currently, the decision tree is the best model for classification and predictions. Similar to a flowchart, a decision tree is a graph that depicts a series of logical process steps as a tree structure. An attribute test is represented as an internal node in a decision tree, a branch as an attribute test result, and a class label as a leaf node. Decision trees are useful for data classification because they can perform this with minimal computational time. The decision tree can be used for handling both continuous and categorical data. Decision trees show which data points are required for accurate classification and predictions [49].

#### 3.4.5. K-Nearest Neighbor

K-Nearest Neighbor (KNN) is a very efficient classification model with less computational cost [50]. It is also known as a lazy learner because it does not require training. The KNN classifier uses the distance metric to measure the similarity to the data points in the neighborhood. The Euclidean distance is used to produce accurate results for KNN [51]. It is not essential to build a model, fine-tune the parameters, nor make any other assumptions. The processing time grows exponentially as the number of independent variables grows.

### 3.5. Deep Learning Models

We also deployed some deep learning architectures in comparison with the machine learning models, which are listed below:

- MobileNet-V2 model: MobileNet-V2 is a lightweight enhanced version of V1 that was designed by Google. It was trained on millions of images using a thousand different categories. Since this is a depthwise separable convolution, it just performs a single convolution on each channel rather than mixing all of the convolutions that are performed. MobileNet-v2 is an architecture that takes up very little time and performs computations very efficiently [52].
- VGG-16 model: VGG16 is a well-known and extensive model for large image datasets, which is used for image object recognition. Karen Simonyan and Andrew Zisserman in their paper [53] “Very Deep Convolutional-Networks for Large-Scale Image Recognition”, from Oxford University, offered VGG16 as a CNN approach. The concept for this model was first described in 2013, and the model itself was submitted in 2014 for the ILSVRC ImageNet Challenge. The final results of large-scale image classification (and object identification) models were evaluated in the ImageNet Wide-Scale Visual Recognition Challenge (ILSVRC). When tested on ImageNet, a database of over 14 million images organized into 1000 classes, the model outperformed 92.7% of the top models currently available.
- DenseNet-121 model: DenseNet-121 was designed to improve testing accuracy in a wide variety of classification tasks by addressing vanishing gradient issues [54]. A lengthy transformation between the input and output layers resulted to some extent in information loss. When compared to ResNet, DenseNet performs better on the measures of accuracy, efficiency, and network size. DenseNet-121 uses 166,280 trainable parameters.
- ResNet-50 model: ResNet-50 is a 50-layer convolutional neural network model that outperforms classification tasks and solves the “vanishing gradient” problem [55]. The ResNet-50 model uses 24,061,192 parameters for training with a categorical loss function and 32 batch sizes, one dense layer with 524544 parameters, and a second dense layer, also called a fully connected layer, with 2056 parameters. Furthermore, 50% dropout is added after the first dense layer. The trainable parameters for deep learning are illustrated in Table 4.

### 3.6. Evaluation Measures

The machine learning and deep learning models’ performances were evaluated using evaluation parameters. A machine learning model was applied to test the data that had previously been unknown to the algorithm to estimate how well it performed on them. Using the test data, the evaluation methods examined the model’s performance and scored it according to its efficiency. To assess how well the models performed in the classification task, the test data were used in conjunction with a training set of the same data. The task of analyzing the performance of an applied model is one of the significant contributions of machine learning. Machine learning models can typically be evaluated based on four basic measures:

- *TP* is the rate that refers to the actual positive class that is predicted positively.
- *TN* is the rate that refers to the correct negative predictions made by the model among all negative samples.
- *FP* is a false positive rate that states the actual negative predictions that are classified as positive by the model.
- *FN* is the rate that states that the data belong to the positive class and are predicted as negative by the machine learning algorithm.

The four basic evaluation measures (accuracy, precision, recall and F1 score) are calculated using the following formulas:

(1) $$Accuracy=\frac{\left(TP+TN\right)}{\left(TP+FP+TN+FN\right)}$$

(2) $$Precision=\frac{\left(TP\right)}{\left(TP+FP\right)}$$

(3) $$Recall=\frac{\left(TP\right)}{\left(TP+FN\right)}$$

(4) $$F1score=2\times \frac{\left(Recall\times Precision\right)}{\left(Recall+Precision\right)}$$

## 4. Results and Discussion

In this section, machine-learning-based experiments using an imbalanced and a balanced dataset of battery making faults are conducted. Five machine learning classifiers were used in this experiment with fine-tuned parameters. Furthermore, pre-trained deep learning models were used to detect the faults in the battery making process in comparison with machine learning. The transfer deep learning experiments were run on a Colab notebook with 16GB RAM and used 20 epochs, 32 batch sizes, a categorical cross-entropy loss function, an AdamX or Adam optimizer, and softmax dense layers to fit the model.

### 4.1. Results of Machine Learning Models

Table 5 represents the results of five machine learning classifiers: LR, DT, KNN, SVM, and RF, with the finely tuned best hyper-parameters. The experiment results showed that the Random Forest (RF) classifier had the highest accuracy of 84% in detecting overall faults in the image dataset. The K-Nearest Neighbor (KNN) classifier came in second with 79% accuracy. The logistic regression achieved 76% accuracy. Other machine learning classifiers also performed well. The RF classifier achieved a 100% precision and F1-score on the burn class and a 99% recall score, as well as a 99% recall on the cover is wrong and normal classes.

The results of machine learning classifiers were also analyzed using an imbalanced dataset. The RF also attained the highest results as compared to other machine learning classifiers, with an accuracy of 65%. The LR achieved 64% accuracy, while the DT only achieved 50% accuracy. Machine learning models could not outperform well using an unbalanced dataset as they outperformed using a balanced dataset after applying SMOTE. We used SMOTE to balance the battery making dataset classes. The machine learning classifier RF performed 19% more accurately on a balanced dataset as compared to the results on the imbalanced dataset.

**Figure 4:**
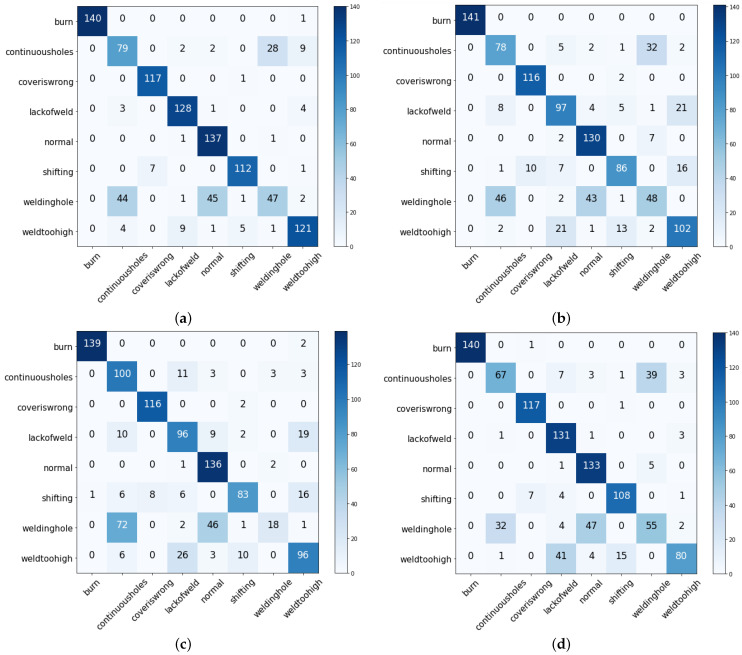
X-axis shows the Actual class and Y-axis shows the Predicted class where (**a**) represents the confusion matrix for Random forest-classifier, (**b**) represents the confusion matrix for Logistic Regression-classifier, (**c**) represents the confusion matrix for Support Vector Machine-classifier, (**d**) represents the confusion matrix for K-Nearest Neighbor-classifier.

**Figure 5:**
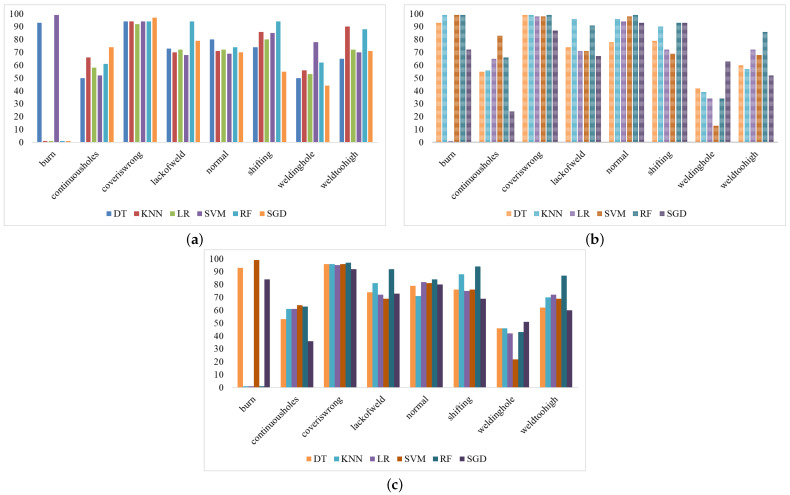
Performance of ML classifiers: (**a**) represents the precision score, (**b**) represents the recall score, and (**c**) represents the F1 score.

The fault detection results also analyzed using the confusion matrix are shown in Figure 4. RF predicted 140 correct predictions out of 141 total predictions, with only one incorrect prediction in the burn class. RF also achieved good prediction in the cover is wrong class with only one wrong prediction. RF achieved 881 correct predictions out of 1055. SVM achieved 784, the LR 798, and the KNN 831 correct predictions. Figure 5a–c show the ML results with various evaluation metrics such as the precision, recall, and F1 score for the eight fault detection classes using a balanced dataset.

### 4.2. Results of Deep Learning Models

Table 6 shows the findings of five deep models based on transfer learning: VGG-16, ResNet-50, MobileNet-V2, CNN, and DenseNet-211. The CNN model achieved the greatest overall fault detection accuracy in the experiments, at 75%. Both the DenseNet-211 and the MobileNet-V2 models were able to perform well with an accuracy of 71% and 71%. Both VGG-16 and ResNet-50 performed poorly, with an accuracy of only 28% and 24%, respectively. The CNN model achieved 94% precision, 97% recall, and a 94% F1 score on the burn class. The above-mentioned results were achieved on the balanced dataset.

The imbalanced dataset was also used to evaluate the efficacy of transfer deep learning models. In terms of total fault detection accuracy, the studies revealed that the CNN model performed best, with a 65% accuracy rate (Table 7). Accuracy was high for the DenseNet-211 and MobileNet-V2 models. Poor results (accuracy of 41%) were achieved by both VGG-16 and ResNet-50. The CNN and MobileNet-V2 models achieved only a 100% precision score on the burn class, while the DenseNet-211 model achieved a 100% recall score on the cover is wrong class. Deep learning did not perform very well in detecting faults in battery making image datasets.

### 4.3. Training and Validation Accuracy Curves

**Figure 6:**
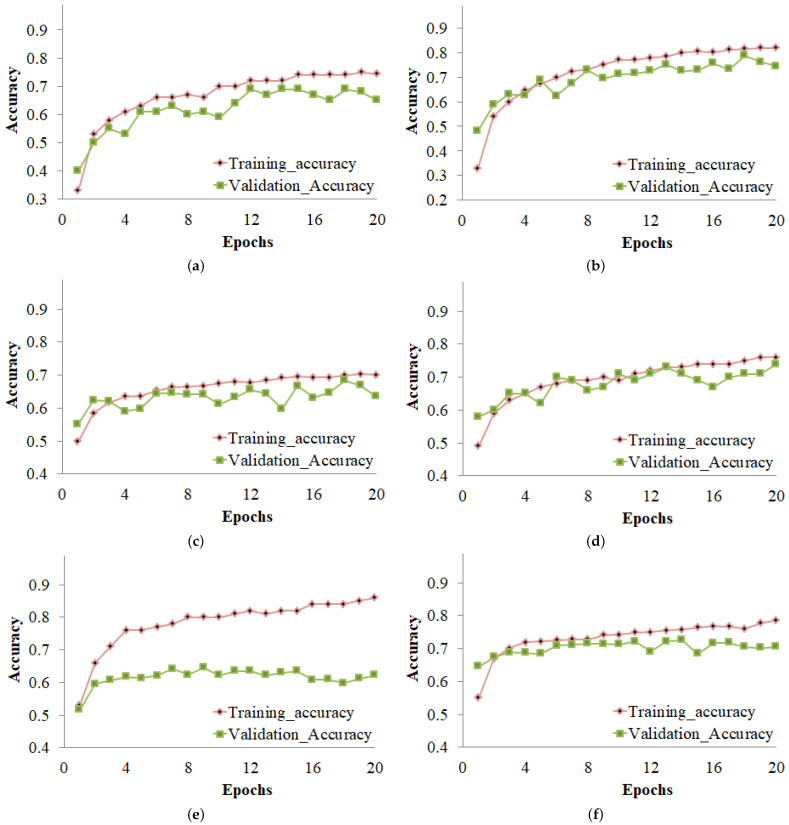
Training and validation accuracy of the (**a**) CNN model using the imbalanced dataset, (**b**) CNN model using the balanced dataset, (**c**) DenseNet-121 model using the imbalanced dataset, (**d**) DenseNet-121 model using the balanced dataset, (**e**) MobileNet-V2 model using the imbalanced dataset, and (**f**) MobileNet-V2 model using the balanced dataset.

Figure 6 illustrates the accuracy of training and validation for several deep learning models on both the imbalanced and balanced datasets. Figure 6a demonstrates that the CNN model achieved its best levels of accuracy during training at Epoch 19 and during validation at Epoch 12. Figure 6b demonstrates that, when employing a balanced dataset, the CNN model achieved its best training accuracy at Epoch 20 and its highest validation accuracy at Epoch 18. Figure 6c–f represent the accuracy for other models.

### 4.4. Training and Validation Loss Curves

**Figure 7:**
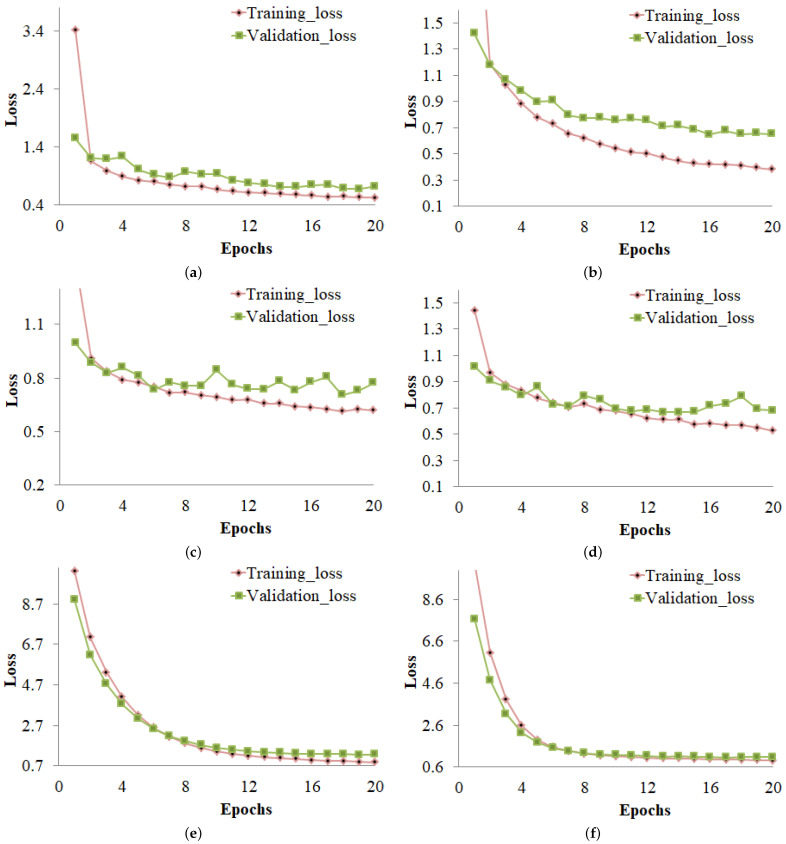
Training and validation loss of (**a**) CNN model using the imbalanced dataset, (**b**) CNN model using the balanced dataset, (**c**) DenseNet-121 model using the imbalanced dataset, (**d**) DenseNet-121 model using the balanced dataset, (**e**) MobileNet-V2 model using the imbalanced dataset, and (**f**) MobileNet-V2 model using the balanced dataset.

The training and validation losses of different deep learning models using the imbalanced and balanced datasets are shown in Figure 7. Figure 7 shows that the CNN model had its highest training loss at Epoch 1 with 3.4130 and lowest at Epoch 20. Figure 7b shows that the CNN model had its highest validation loss at Epoch 1 with 1.5459 and lowest at Epoch 18 with 0.6796 using an imbalanced dataset.

Figure 7 illustrates the training and validation losses of various deep learning models on both the imbalanced and balanced datasets. The training loss for the CNN model was highest at Epoch 1 with 2.8841 and lowest at Epoch 20, as shown in Figure 7a. Figure 7b demonstrates that, after using a balanced dataset, the CNN model’s validation loss was highest at Epoch 1 with 1.4192 and lowest at Epoch 18 with 0.6515. The losses of other models are shown in Figure 7c–f. The loss curves show that the deep learning models performed best with a balanced dataset with the lowest training and validation loss.

To perform an extensive comparison, we also used several variants of the CNN in comparison with our approach. We deployed the variant of the CNN with a change in the number of thearchitecture hyper-parameter values, as shown in Table 8. All CNN variants were common in the first layer and last layers, as well as the compile and fitting settings.

Table 9 shows the results of the fine-tuned CNN variants, and according to the results, all variants were approximately similar in their accuracies, as CNN-1, CNN-2, and CNN-3 achieved 77, 78, and 76 accuracy scores, respectively.

We also checked the computational complexity of the Machine Learning (ML) and Deep Learning (DL) models in terms of the computational time. We measured the time (in seconds) for each model that it took for training and testing. Table 10 shows the computational time in seconds for both the ML and DL models. ML models were very low in computational cost as compared to the DL models because of their simple architectures, while the deep learning model architectures are complex and took more time for training.

### 4.5. K-Fold Cross-Validation Results

The machine learning results were also evaluated by applying K-fold cross-validation (10-fold and 5-fold) on a balanced dataset and are presented in Table 11. LR and RF achieved the highest accuracy on 10-fold and 5-fold cross-validation. LR achieved 81.897% accuracy with a 10-fold standard deviation rate of 0.0255. The Decision Tree achieved 71.246% accuracy with 10-fold and 71.275 with 5-fold validation, which is the lowest in machine learning, and also a 0.0336 standard deviation.

### 4.6. Comparison of Other State-of-the-Art Models with Our Model

We compared our model with the state-of-the-art models on the same battery fault detection dataset. Support vector machine with the RBF kernel [56], the extra tree classifier [57] with 300 total estimators, random state 5, maximum of depth 300, and random forest [58] all yielded unsatisfactory results. Comparisons with our model were made using the accuracy, precision, recall, and the F1 score. All of the metrics showed that our model performed the best. Table 12 shows the comparison with state-of-the-art approaches.

## 5. Conclusions

The aim of this work was to automatically detect battery faults from a battery image dataset using machine learning and image processing. The experimentation dataset contained eight classes and was highly imbalanced. The SMOTE was used to balance the dataset. Machine learning models were used to detect faults in batteries on balanced and imbalanced datasets in order to evaluate the performance. The deep learning models were used to detect faults in comparison with machine learning. Furthermore, 10-fold cross-dataset validation and 5-fold cross-validation were performed with machine learning models to ensure the algorithms’ reliability and validity. Our best results were with the RF model, which performed well with 0.84 accuracy scores using our proposed CNN features and SMOTE over-sampled data. There are several conclusions from the studies: First, feature extraction is one of the important things to improve the accuracy of learning models, and we used the CNN, which extracts the worthy features from the image dataset to improve the performance of the learning models. Second, an imbalanced dataset can cause model over-fitting for majority class data, and to resolve this issue, data balancing is important. For this, we used SMOTE to generate artificial data for minority class data. Third, we concluded that image data generate a large feature space, which is suitable for linear models such as LR and tree-based ensemble models. Both achieved significant results because of the large feature set. Finally, we also concluded that deep learning models require a large dataset to achieve significant results, and this study’s dataset was not enough for the deep learning models, which is also a limitation of the study. In future work, we will collect more images and will create a large dataset. We will resolve the imbalanced dataset problem by collecting more images for the minority class category. In this study, we took images only from a single angle (from above), but probably, other angles would be beneficial for increasing the quality of the results. In our future work, we will work on this limitation. A transfer learning approach will also be used in our future work to detect battery faults.

## Figures and Tables

**Table 1:** Brief summary of the aims and limitations of previous studies on fault detection.

Ref	Models	Dataset	Aim	Limitations
[15]	SqueezeNet	Welding defect images	Automatic inspection of laser welding defects using deep learning.	The experiments in the study used a large dataset, but some classes were imbalanced. They caused over-fitting of the model.
[16]	VGG model	Laser welding images	Given large datasets, a pre-trained SqueezeNet was utilized to detect the welding fault of the safety vent on the power battery.	The study solved the two and three classification problems and did not use machine learning to detect the faults. Furthermore, it did not conduct cross-dataset experiments.
[17]	First-order RC model	Lithium cells	The basic aim was to improve the accuracy of detecting external short-circuits in lithium batteries using the DPSO and RC algorithms.	These methods were only used to identify battery defects and their levels; they were unable to detect accidents in a responsible way and may have missed certain battery faults.
[20]	SVM	Battery cells	SVM-based estimation of the state-of-health and prediction of the remaining useful life of lithium batteries.	The results were unsatisfactory.
[22]	mRVM	Battery packs	Detection of common battery pack defects using machine learning models.	The likelihood of effective fault isolation was relatively low.
[24]	SVM	Battery data	Automated and real-time internal short-circuit fault detection using powerful machine learning models.	The deep transfer learning approach was not used.
[26]	Sample entropy	Battery system	To diagnose battery faults in electric vehicles, a unique approach based on sample entropy was proposed.	The study did not use machine learning to diagnose the defects.
[27]	Improved Res2Net	Safety vent welding defect images	An effective multi-scale attention semantic segmentation method for power battery laser fault detection.	The study did not use any feature extractors or machine learning to diagnose the defects.

**Table 2:** Splitting the datasets into training and testing.

Datasets	Training	Testing	Total
Imbalanced dataset	3175	561	3736
Balanced dataset	5977	1055	7032

**Table 3:** Hyper-Parameters Setting for Machine learning.

Models	Hyper-Parameters
LR	random_state = 200, solver = “sag”, multi_class = “multinomial”, C = 3.0
RF	n_estimators = 300, random_state = 5, max_depth = 300
KNN	Default_parameters
DT	max_depth = 300
SVM	kernel = “poly”, C = 3.0, random_state = 500

**Table 4:** Trainable parameters for deep learning and their setting.

Models	Parameters	Settings
CNN	5,398,998	loss= “categorical_crossentropy”, lr = 0.001, optimizer = adam, batch_size = 32
DenseNet-121	166,280	loss = “categorical_crossentropy”, dropout = 0.001, optimizer = adam, batch_size = 32
VGG-16	137,496	loss = “categorical_crossentropy”, dropout = 0.001, optimizer = adam, batch_size=32
ResNet-50	24,061,192	loss = “categorical_crossentropy”, dropout = 0.001, optimizer = adam, batch_size = 32
MobileNet-V2	662,536	loss = “categorical_crossentropy”, neurons = 512, dropout = 0.3, lr = 0.001, optimizer = Adamax

**Table 5:** Performance of machine learning models on the imbalanced and balanced datasets.

	Imbalanced Dataset					Balanced Dataset			
**Classifiers**	**Accuracy**	**Faults**	**Precision**	**Recall**	**F1 Score**	**Accuracy**	**Precision**	**Recall**	**F1 Score**
DT	50	burn	53	47	47	73	93	93	93
		continuous holes	50	55	52		50	55	53
		cover is wrong	97	88	92		94	99	96
		lack of weld	18	15	16		73	74	74
		normal	52	54	53		80	78	79
		shifting	28	19	18		74	79	76
		welding hole	38	35	37		50	42	46
		weld too high	64	64	64		65	60	62
KNN	61	burn	100	68	81	79	100	99	100
		continuous holes	62	66	64		66	56	61
		cover is wrong	97	100	99		94	99	96
		lack of weld	45	47	46		70	96	81
		normal	50	64	56		71	96	71
		shifting	61	35	45		86	90	88
		welding hole	45	41	43		56	39	46
		weld too high	73	70	71		90	57	70
LR	64	burn	100	74	85	76	100	100	100
		continuous holes	65	67	66		58	65	61
		cover is wrong	97	94	96		92	98	95
		lack of weld	42	33	37		72	71	72
		normal	59	78	68		72	94	82
		shifting	50	35	42		80	72	75
		welding hole	49	38	43		53	34	42
		weld too high	69	84	76		72	72	72
SVM	63	burn	100	74	85	74	99	99	99
		continuous holes	59	86	70		52	83	64
		cover is wrong	97	100	97		94	98	96
		lack of weld	67	10	17		68	71	69
		normal	57	99	72		69	98	81
		shifting	67	6	12		85	69	76
		welding hole	55	10	16		78	13	22
		weld too high	64	89	74		70	68	69
RF	65	burn	100	79	88	84	100	99	100
		continuous holes	66	69	68		61	66	63
		cover is wrong	97	100	99		94	99	97
		lack of weld	53	25	34		94	91	92
		normal	58	91	71		74	99	84
		shifting	75	10	17		94	93	94
		welding hole	56	35	43		62	34	43
		weld too high	64	90	75		88	86	87

**Table 6:** Results of deep transfer learning using the balanced dataset.

Models	Accuracy%	Faults	Precision%	Recall%	F1 Score%
CNN	75	burn	94	97	94
		continuous holes	81	45	54
		cover is wrong	96	86	91
		lack of weld	86	58	69
		normal	77	67	72
		shifting	66	84	74
		welding hole	47	79	59
		weld too high	73	82	77
DenseNet-211	74	burn	95	87	91
		continuous holes	69	71	70
		cover is wrong	85	95	90
		lack of weld	72	63	67
		normal	68	91	78
		shifting	69	65	67
		welding hole	57	38	45
		weld too high	69	76	73
VGG-16	28	burn	99	98	98
		continuous holes	16	100	27
		cover is wrong	0	0	0
		lack of weld	0	0	0
		normal	0	0	0
		shifting	0	0	0
		welding hole	0	0	0
		weld too high	0	0	0
ResNet-50	24	burn	33	93	48
		continuous holes	0	0	0
		cover is wrong	38	30	33
		lack of weld	0	0	0
		normal	50	1	2
		shifting	15	74	25
		welding hole	0	0	0
		weld too high	0	0	0
MobileNet-V2	71	burn	95	93	94
		continuous holes	60	85	70
		cover is wrong	88	94	91
		lack of weld	65	56	60
		normal	69	63	66
		shifting	63	62	63
		welding hole	44	24	31
		weld too high	67	79	73

**Table 7:** Results of deep transfer learning using the imbalanced dataset.

Models	Accuracy%	Faults	Precision%	Recall%	F1 Score%
CNN	65	burn	100	96	98
		continuous holes	69	62	65
		cover is wrong	96	85	90
		lack of weld	56	56	56
		normal	53	29	38
		shifting	96	62	75
		welding hole	47	61	53
		weld too high	72	86	78
DenseNet-211	65	burn	92	96	94
		continuous holes	65	66	65
		cover is wrong	93	100	96
		lack of weld	48	67	56
		normal	52	94	67
		shifting	53	92	67
		welding hole	56	29	38
		weld too high	86	56	67
VGG-16	41	burn	0	0	0
		continuous holes	33	99	49
		cover is wrong	0	0	0
		lack of weld	12	2	4
		normal	0	0	0
		shifting	0	0	0
		welding hole	0	0	0
		weld too high	64	89	74
ResNet-50	41	burn	100	46	63
		continuous holes	24	47	32
		cover is wrong	68	100	81
		lack of weld	39	21	27
		normal	56	68	61
		shifting	81	35	49
		welding hole	48	52	50
		weld too high	100	01	02
MobileNet-V2	62	burn	100	88	93
		continuous holes	58	77	93
		cover is wrong	93	100	96
		lack of weld	60	49	54
		normal	46	44	45
		shifting	81	59	69
		welding hole	42	31	35
		weld too high	75	83	79

**Table 8:** Different fine-tuned variants of the CNN used to perform the experiments.

CNN 1	
Sequential () preprocessing.Rescaling (1./255, input_shape = (150, 150, 3))) Conv2D (filters = 32, kernel_size = (3, 3), activation = “relu”, kernel_initializer = “he_normal”) MaxPooling2D (pool_size = (2, 2)) Dropout (0.1) Conv2D (filters = 64,kernel_size = (3, 3), activation = “relu”, kernel_initializer = “he_normal”) MaxPooling2D (pool_size = (2, 2)) Dropout (0.2) Conv2D (filters = 256,kernel_size = (3, 3), activation = “relu”, kernel_initializer = “he_normal”) MaxPooling2D (pool_size = (2, 2)) Dropout (0.2) Flatten () Dense (256, activation = “relu”) Dense (512,activation = “relu”) Dense (256, “relu”)	
**CNN 2**	**CNN 3**
Sequential () preprocessing.Rescaling (1./255, input_shape = (150, 150, 3)) Conv2D (filters = 32, kernel_size = (3, 3), activation = “relu”) MaxPooling2D (pool_size = (2, 2)) Conv2D (filters = 64, kernel_size = (3, 3), activation = “relu”) MaxPooling2D (pool_size = (2, 2)) Conv2D (filters = 64, kernel_size = (3, 3), activation = “relu”) MaxPooling2D (pool_size = (2, 2)) Conv2D (filters = 64, kernel_size = (3, 3), activation = “relu”) MaxPooling2D (pool_size = (2, 2)) Dropout (0.2) Flatten ()) Dropout (0.5) Dense (128, activation = “relu”) Dense (256, activation = “relu”)) Dense (512, activation = “relu”))	Sequential () preprocessing.Rescaling (1./255, input_shape = (150, 150, 3)) Conv2D (filters = 32, kernel_size = (3,3), activation = “relu”) Conv2D (filters = 32, kernel_size = (3, 3), activation = “relu”) Conv2D (filters = 32, kernel_size = (3, 3), activation = “relu”) Conv2D (filters = 32, kernel_size = (3, 3), activation = “relu”) Conv2D (filters = 32, kernel_size = (3, 3), activation = “relu”) MaxPooling2D () Dropout (0.2)) Flatten ()) Dropout (0.5) Dense (128, activation = “relu”) Dense (256, activation = “relu”))
Dense (8, “softmax”)) compile(loss = “categorical_crossentropy”, optimizer = “Adam”, metrics = [“accuracy”])	

**Table 9:** Results of the fine-tuned CNN models.

Models	Accuracy	Parameters	Faults	Precision	Recall	F1 Score
Fine-tuned CNN 1	77	21,665,992	burn	93	99	95
			continuous holes	71	50	58
			cover is wrong	94	96	95
			lack of weld	77	60	68
			normal	69	94	79
			shifting	84	78	81
			welding hole	56	52	54
			weld too high	70	87	78
Fine-tuned CNN 2	78	889,672	burn	96	99	98
			continuous holes	65	76	70
			cover is wrong	96	98	97
			lack of weld	83	71	76
			normal	77	81	79
			shifting	85	76	80
			welding hole	49	37	42
			weld too high	68	85	76
Fine-tuned CNN 3	76	23,113,096	burn	96	94	95
			continuous holes	62	64	63
			cover is wrong	91	93	92
			lack of weld	83	81	82
			normal	70	85	82
			shifting	79	85	82
			welding hole	49	43	46
			weld too high	78	78	78

**Table 10:** Computational complexity in terms of time for learning models.

ML Model	Time	DL Model	Time
DT	9.13	DenseNet121	231
KNN	0.69	ResNet50	700
LR	38.29	VGG16	360
SVM	18.58	MobileNetV2	100
RF	35.65	Fine-tuned CNN 1	122
SGD	16.54	Fine-tuned CNN 2	100
		Fine-tuned CNN 3	361

**Table 11:** K-fold cross-validation results.

Models	10-Fold Accuracy	05-Fold Accuracy
DT	71.246 ± 0.0336	71.275 ± 0.0327
KNN	76.008 ± 0.0310	74.999 ± 0.0321
LR	81.897 ± 0.0255	80.930 ± 0.0178
SVM	80.588 ± 0.0248	79.735 ± 0.0148
RF	81.285 ± 0.0304	80.218 ± 0.0340

**Table 12:** Comparison of other state-of-the-art models with our model.

Models	Accuracy%	Precision%	Recall%	F1 Score%
Modified VGG-16 [15]	61	62	61	59
ETC [57]	67	66	67	65
RF [58]	63	62	63	61
SVM (RBF kernel) [56]	66	67	67	66
SVM [59]	24	13	28	13
**Our study**	**84**	**71**	**84**	**70**

## Data Availability

Not applicable.
